# Family quality of life among families who have children with mild intellectual disability associated with mild autism spectrum disorder

**DOI:** 10.1590/0004-282X-ANP-2020-0537

**Published:** 2022-03-14

**Authors:** Marcela Cesaretti Borilli, Carla Maria Ramos Germano, Lucimar Retto da Silva de Avó, Rui Fernando Pilotto, Débora Gusmão Melo

**Affiliations:** 1 Universidade Federal de São Carlos, Programa de Pós-Graduação em Enfermagem, São Carlos SP, Brazil.; 2 Universidade Federal de São Carlos, Departamento de Medicina, São Carlos SP, Brazil.; 3 Instituto Nacional de Genética Médica Populacional, Porto Alegre RS, Brazil.; 4 Universidade Federal do Paraná, Departamento de Genética, Curitiba PR, Brazil.

**Keywords:** Intellectual Disability, Autism Spectrum Disorder, Quality of Life, Family, Family Relations, Brazil, Deficiência Intelectual, Transtorno do Espectro Autista, Qualidade de Vida, Família, Relações Familiares, Brasil

## Abstract

**Background:**

Intellectual disability (ID) and autism spectrum disorder (ASD) are often concomitant childhood developmental disorders. These disorders can alter family quality of life (FQoL).

**Objective:**

To investigate FQoL among families who have children with mild ID, associated with mild ASD.

**Methods:**

Cross-sectional descriptive study with 69 families who have children with mild ID and ASD, ranging from six to 16 years old, and who were provided with disability-related services in Brazil. Data were collected using a family sociodemographic questionnaire, an ID and ASD personal profile form, the Barthel index for activities of daily living and the Beach Center FQoL scale.

**Results:**

People with ID and ASD had an average score of 88.2±11.5 in the Barthel index, thus indicating moderate dependency in basic activities of daily living. The average total FQoL score (3.56±0.34) was lower than the scores for the “family interaction” (3.91±0.42; p<0.001), “parenting” (3.79±0.35; p<0.001) and “disability-related support” (3.98±0.16; p<0.001) domains; and higher than the scores for the “physical/material well-being” (3.19±0.64; p<0.001) and “emotional wellbeing” (2.75±0.62; p<0.001) domains. Parents’ marital condition, monthly family income, family religious practice and effective communication skills among the people with ID and ASD were predictors for FQoL (R^2^=0.407; p<0.001).

**Conclusions:**

FQoL was sustained through factors such as family interaction and parents’ care for their children. Improving families’ emotional wellbeing and physical and material conditions is likely to positively affect the FQoL of these families.

## RESUMO

**Antecedentes:**

Deficiência intelectual (DI) e transtorno do espectro do autismo (TEA) são distúrbios do desenvolvimento infantil frequentemente concomitantes que podem impactar na qualidade de vida familiar (QVF).

**Objetivo:**

Esta pesquisa avaliou a QVF entre famílias que têm filhos com DI leve associada a TEA leve.

**Métodos:**

Pesquisa transversal e descritiva, que investigou 69 famílias com filhos com DI e TEA leves, com idades entre seis e 16 anos, que recebiam serviços relacionados à deficiência no Brasil. Os dados foram coletados por meio de formulário sociodemográfico, formulário de perfil da pessoa com DI e TEA, o índice de funcionalidade de Barthel e a Escala de QVF do Beach Center.

**Resultados:**

Os indivíduos com DI e TEA obtiveram pontuação média de 88,2±11,5 no índice de Barthel, o que indicou dependência moderada nas atividades básicas de vida diária. O escore médio da QVF total (3,56±0,34) foi menor que os escores dos domínios “interação familiar” (3,91±0,42; p<0,001), “cuidados dos pais com os filhos” (3,79±0,35; p<0,001) e “apoio ao deficiente” (3,98±0,16; p<0,001), e maior que os escores dos domínios “bem-estar físico-material” (3,19±0,64; p<0,001) e “bem-estar emocional” (2,75±0,62; p<0,001). Condição marital dos pais, renda mensal, prática religiosa e comunicação adequada dos indivíduos com DI e TEA foram preditores da QVF (R^2^=0,407; p<0,001).

**Conclusões:**

A QVF foi sustentada por aspectos como a interação familiar e o cuidado dos pais com os filhos. Melhorar o bem-estar emocional e as condições físicas e materiais familiares provavelmente afetará positivamente a qualidade de vida dessas famílias.

## INTRODUCTION

Intellectual disability (ID) is a developmental disorder characterized by impaired general mental abilities. It results in deficits of both intellectual and adaptive functioning, such that individuals cannot achieve the standards of personal independence and social responsibility in one or more aspects of their daily lives^1^. ID has a global frequency of about 1 to 3%, varying according to age, and it is more common among males^2^. It has been estimated that 1.4% of the Brazilian population has some degree of ID^3^. ID can be classified as mild, moderate, severe or profound. Approximately 85% of people who have ID have mild ID. These individuals are characterized as not benefitting from the instruction that they receive for higher performance in their academic and working lives, having flaws in their processes of abstract conceptualization and fluctuating attention, but having autonomy in basic activities of daily life^1^.

Autism spectrum disorder (ASD) is a developmental disorder characterized by persistent impairment in social reciprocal communication and social interaction, and also restricted and repetitive patterns of behavior, interests or activities^1^. ASD has an estimated global frequency of around 1 to 2%, but it is three to four times more common among males^1,4^. It can be classified as mild, moderate or severe, and the criterion adopted for assessing severity relates to the amount of support needed to address a person's needs, considering their difficulties^1^.

ASD and ID are common comorbidities^2,4^. While at least 10% of individuals with ID have ASD, about 50 to 80% of individuals with ASD have some degree of ID^5,6^. Caring for individuals with ID and ASD often results in an emotional and financial burden on their families^7–9^. Having a family member with a disability alters the family's dynamics and quality of life^10,11^.

Families can be defined as groups of people who are closely involved in the day-to-day affairs of the household and support each other regularly; whether related by blood, marriage or close personal relationship^12^. In this context, family quality of life (FQoL) can be understood as family wellbeing in a dynamic sense, subjectively perceived and informed by its own members, contemplating interactions between individual and family needs^13,14^. Research on the FQoL of families who have members with ID and/or ASD has been explored with the aim of shaping public policies that encourage care in this area, and also to contribute to evaluations on services and clinical interventions^14–16^.

The present study had the aim of investigating FQoL in a sample of Brazilian families who have children with mild ID in association with mild ASD.

## METHODS

### Study design and setting

This was a descriptive cross-sectional study that was developed with support from the Association of Parents and Friends of Exceptional People of São Carlos (Associação de Pais e Amigos de Excepcionais de São Carlos, APAE). São Carlos is a city located in the state of São Paulo, in southeastern Brazil, with approximately 250,000 inhabitants. In 2010, its human development index was 0.8059^3^. APAE São Carlos was founded in 1962 and currently serves about 800 individuals with ID and/or ASD, offering specialized education and support.

This study was approved by the Human Research Ethics Committee of the Universidade Federal de São Carlos and participation was authorized through signing an informed consent declaration.

### Participants

This study was developed using a purposeful convenience sample^17^, consisting of families who had children with mild ID in association with mild ASD, and who had links to APAE São Carlos. The inclusion criteria were: (1) age range of the child between 6 and 16 years; (2) clinical diagnosis of mild ID confirmed through the Wechsler Intelligence Scale for Children (WISC-IV)^18^; and (3) clinical diagnosis of mild ASD confirmed through the Childhood Autism Rating Scale (CARS)^19^. We identified 69 families that met these inclusion criteria. All of these families were invited and agreed to participate in the study.

### Data collection

Data collection was carried out using printed forms and was done individually by a single researcher in face-to-face situations with one interviewee at a time, between July 2018 and May 2019. Regarding the informant, in 56 families (81%), this was the mother; in six families (9%), the father; in four families (6%), an uncle or aunt; and in three families (4%), the grandfather or grandmother.

The data collection instruments were the “family sociodemographic profile” and the “ID and ASD personal profile” forms, the Barthel index and the Beach Center FQoL scale. The “family sociodemographic profile” form was designed for this study and asked for information on the number of people in the household, monthly family income, receipt of social benefits, supplemental health insurance plan, religion, parents’ marital status, maternal and paternal education, parents’ jobs and the number of siblings. The “ID and ASD person profile” form was also designed for the present study and asked for information on these individuals’ gender, age, educational level, communication skills and autonomy indoors.

The Barthel index belongs to the field of assessment of basic activities of daily living and assesses the level of independence in relation to ten activities. The total score of this instrument ranges from 0 to 100, such that a score of 0–20 indicates total dependence; 21–60, severe dependence; 61–90, moderate dependence; 91–99, mild dependence; and 100, independence^20^.

To assess FQoL, the Beach Center Family Quality of Life Scale (BCFQoLS) was used^21^, in its version translated into Portuguese^22^. This instrument consists of a 25-item, five-domain questionnaire (parenting, family interaction, emotional wellbeing, physical/material wellbeing and disability-related support), with five possible answers on a Likert scale to measure satisfaction. The sum of points obtained in each domain represents the FQoL grand total^21^. The scores for each BCFQoLS domain, along with the FQoL grand total, are transformed into a quinary ratio, and scores ≥4.0 indicate satisfaction^21,23^.

### Data analysis

The findings were presented as the mean, median and standard deviation (SD), or absolute frequency and percentage, according to the type of variable. The internal consistency of the BCFQoLS and Barthel index was assessed by means of Cronbach's alpha coefficient, and values above 0.70 were considered ideal.

The FQoL grand total was verified using the Kolmogorov-Smirnov test and was found to present normal distribution (D=0.09195; p=0.57223); therefore, parametric statistical tests were used. Differences in means between the several BCFQoLS domains and the FQoL grand total were determined using the paired Student's *t*-test. The effect of sociodemographic factors on the total FQoL level was determined by means of the independent (unpaired) Student's *t*-test or using analysis of variance (ANOVA) with the Tukey post-test, according to the number of variables analyzed.

Binary correlations between the different BCFQoLS domains and the FQoL grand total were determined using the Pearson linear correlation. The Pearson or Spearman correlation was used to verify the binary correlation between the total FQoL and each of the other variables of the study. The intensity of the correlation coefficient (r) was taken to be a weak correlation when it was between 0 and 0.3, a moderate correlation when between 0.3 and 0.6 and a strong correlation when above 0.6.

Multiple linear regression models were used for multiple correlation analysis in two ways: (1) enter - variables that were significant in the binary correlation with p<0.05 were all included in the model at the same time, to assess their contribution to the FQoL grand total; (2) stepwise - non-contributing variables were excluded by means of the step-by-step statistical program, to identify the most significant correlations. To verify the quality of the adjusted model, the coefficient of multiple determination (R^2^) was calculated and the significance of the model was determined using ANOVA.

The significance level adopted was 5%. All analyses were performed using the JASP 0.10.2 software (https://jasp-stats.org/).

## RESULTS

### Descriptive results

The sample was characterized by a predominance of families comprising three or more people (97%; n=67), with an average monthly family income of R$ 2,806.52±1,493.75 (currency conversion: $ 1.00=R$ 5.67 on March 10, 2021). Most families were only using the Brazilian National Health System (57%; n=39) and were not receiving any social benefits (81%; n=56).

The mothers were on average 39±5.3 years old, with a minimum age of 29 and a maximum of 56 years; there was incomplete information about one mother. The fathers were on average 42±6 years old, with a minimum age of 32 and a maximum of 64 years. The individuals with ID and ASD were on average 9.5±2.6 years old; 85% (n=60) were male; and 62% (n=43) were literate. No person with ID or ASD had chronic health problems requiring regular use of medication.

Table 1 shows the results regarding the BCFQoLS and Barthel index. The result for the Barthel index was 88.2±11.5, which was compatible with moderate dependence. The average score for the FQoL grand total was 3.56±0.34, i.e. lower than what is considered satisfactory.

**Table 1:** Results from the Beach Center Family Quality of Life Scale and Barthel index among the families investigated that had children with mild intellectual disability and autism spectrum disorder (n=69).

	Domains					FQoL grand total	Barthel index
	Family interaction	Parenting	Emotional wellbeing	Physical/material wellbeing	Disability-related support		
Mean	3.91	3.79	2.75	3.19	3.98	3.56	88.2
SD	0.42	0.35	0.62	0.64	0.16	0.34	11.5
Median	4.00	3.83	2.75	3.20	4.00	3.60	90.0
Minimum	2.33	2.33	2.00	2.00	3.50	2.48	45.0
Maximum	4.83	4.83	4.00	4.80	4.50	4.48	100.0
Cronbach's alpha	0.8520	0.7483	0.7707	0.8190	0.3402	0.8927	0.8189

BCFQoLS: Beach Center Family Quality of Life Scale; ID: intellectual disability; ASD: autism spectrum disorder; SD: standard deviation; FQoL: Family Quality of Life.

The differences in means between the various BCFQoLS domains and the FQoL grand total level are shown in Table 2. The mean value for the FQoL grand total was significantly higher than the scores obtained in the “emotional well-being” (2.75±0.62; p<0.001) and “physical/material well-being” (3.19±0.64; p<0.001) domains; and significantly lower than the scores obtained in the “family interaction” (3.91±0.42; p<0.001), “parenting” (3.79±0.35; p<0.001) and “disability-related support” (3.98±0.16; p<0.001) domains.

**Table 2:** Mean differences and binary correlations across the multiple Beach Center Family Quality of Life Scale domains and the Family Quality of Life grand total in the sample investigated (n=69).

Pairwise comparison: FQoL (grand total) and domains		t	*p-value	r	#p-value
FQoL (grand total)	Family interaction	-11.875	<0.001	0.816	<0.001
FQoL (grand total)	Parenting	-9.165	<0.001	0.824	<0.001
FQoL (grand total)	Emotional wellbeing	14.961	<0.001	0.707	<0.001
FQoL (grand total)	Physical/material wellbeing	7.261	<0.001	0.809	<0.001
FQoL (grand total)	Disability-related support	-11.252	<0.001	0.423	<0.001

BCFQoLS: Beach Center Family Quality of Life Scale; FQoL: Family Quality of Life

* Paired-sample Student's *t*-test; #Pearson's linear correlation.

The “family interaction” (r=0.816; p<0.001), “parenting” (r=0.824; p<0.001), “emotional well-being” (r=0.707; p<0.001) and “physical/material well-being” (r=0.809; p<0.001) domains were strongly correlated with the FQoL grand total (Table 2). In addition, the “family interaction” and “parenting” domains presented a strong correlation with each other (r=0.693; p<0.001).

### Relationships between the characteristics of families and children and satisfaction with family quality of life

The relationships between sociodemographic and family characteristics and FQoL are presented in Table 3. Differences in the mean distribution of FQoL were identified in relation to family income (p=0.021), access to supplemental health insurance (p=0.002), receiving social benefits (p=0.018), religious practice (p=0.011) and parents’ marital status (p<0.001). Tukey's post-test showed that, regarding family income, there was a difference between the “up to R$ 2,000.00” and “between R$ 5,000.00 and R$ 10,000.00” groups (p=0.013). Regarding access to supplemental health insurance, the difference observed was between the groups “no family member has a supplemental health insurance plan” and “every family member has a supplemental health insurance plan” (p=0.002).

**Table 3:** Family Quality of Life grand total distribution according to the sociodemographic and family characteristics of the families investigated that had children with mild intellectual disability and autism spectrum disorder (n=69).

Demographic and family variables		FQoL grand total±SD	p-value
Family income (R$)†	Up to 2,000 (n=25)	3.44±0.27	0.021*
	2,000 to 3,000 (n=16)	3.56±0.41	
	3,000 to 5,000 (n=21)	3.60±0.33	
	5,000 to 10,000 (n=7)	3.87±0.17	
Supplemental health insurance plan	No family member is covered by supplemental health insurance (n=39)	3.44±0.36	0.002*
	Only the individuals with ID/ASD are covered by supplemental health insurance (n=13)	3.65±0.21	
	The whole family is covered by supplemental health insurance (n=17)	3.77±0.24	
Social benefits	Does not gain social benefit (n=56)	3.61±0.31	0.018**
	Gains social benefit (n=13)	3.36±0.38	
Religion	Does not profess a religion (n=14)	3.36±0.35	0.011**
	Professes a religion (n=55)	3.61±0.32	
Parents’ marital status	Divorced or separated (n=20)	3.33±0.40	<0.001**
	Married or living together (n=49)	3.65±0.26	
Mother's job	Works outside the home, full-time or part-time (n=29)	3.51±0.27	0.195**
	Does not work outside the home (n=39)	3.62±0.36	
Mother's educational level	Primary level incomplete (n=6)	3.57±0.32	0.186*
	Primary level complete or secondary level incomplete (n=18)	3.45±0.47	
	Secondary level complete or tertiary level incomplete/complete (n=44)	3.62±0.25	
Father's educational level	Primary level incomplete (n=5)	3.42±0.26	0.176*
	Primary level complete or secondary level incomplete (n=8)	3.35±0.56	
	Secondary level complete or tertiary level incomplete/complete (n=48)	3.60±0.30	
	Postgraduate studies (n=8)	3.64±0.28	
Number of siblings	None (n=33)	3.48±0.26	0.116*
	One sibling (n=26)	3.66±0.35	
	Two siblings (n=10)	3.58±0.47	

FQoL: Family Quality of Life; ID: intellectual disability; ASD: autism spectrum disorder; SD: standard deviation

† The Brazilian Real (R$) is the official currency of Brazil: U$ 1.00=R$ 5.67, on March 10, 2021

* ANOVA

** independent (unpaired) Student's *t*-test.

The relationship of the individual and clinical characteristics of people with ID and ASD with regard to the FQoL is presented in Table 4. Differences in the mean distribution of the FQoL were identified according to the presence of effective communication (p=0.024).

**Table 4:** Family Quality of Life grand total distribution according to the characteristics of the individuals investigated with mild intellectual disability and autism spectrum disorder (n=69).

Personal and clinical variables of the individuals with mild ASD and ID		FQoL grand total±SD	p-value
Age group (3 categories)	6 to 8 years (n=27)	3.58±0.23	0.413*
	8 to 12 years (n=29)	3.60±0.41	
	12 to 16 years (n=13)	3.45±0.35	
Sex	Female (n=9)	3.54±0.32	0.862**
	Male (n=60)	3.56±0.34	
Educational level	Literate (n=43)	3.61±0.33	0.096**
	Illiterate (n=26)	3.47±0.34	
Effective communication	Yes (n=52)	3.61±0.33	0.024**
	No (n=17)	3.40±0.33	
Autonomy for ADL indoors	No autonomy (n=46)	3.54±0.33	0.372**
	Total autonomy (n=23)	3.61±0.36	

FQoL: Family Quality of Life; ID: intellectual disability; ASD: autism spectrum disorder; SD: standard deviation

* ANOVA

** independent (unpaired) Student's *t*-test.

Eight of the variables investigated correlated with the FQoL grand total and were included in the multiple linear regression model: family income, access to supplemental health insurance, receiving social benefits, religious practice, parents’ marital status, paternal educational level, effective communication and educational level of individuals with ID and ASD. The multiple correlation analysis showed that the parents’ marital status, family income, effective communication and religious practice were predictors of the FQoL grand total (Table 5). The coefficient of determination for this final model was R^2^=0.407, which indicated that the model explained 40.7% of the variability found in the FQoL grand total results (p<0.001).

**Table 5:** Multiple correlations of Family Quality of Life grand total with the other variables, calculated by means of the linear regression method (n=69).

			Unstandardized β coefficients	Standardized β coefficients	p-value	R^2^	ANOVA p-value
Multiple correlation – ‘enter’ method							
	FQoL	Constant	2.833		<0.001	0.420	<0.001
		Family income	5.220e^-5^	0.232	0.076		
		Supplemental health insurance plan	0.032	0.081	0.534		
		Social benefit	0.026	0.031	0.797		
		Religion	0.180	0.216	0.040		
		Parents’ marital status	0.203	0.275	0.031		
		Father's educational level	0.043	0.091	0.413		
		Number of siblings	0.033	0.070	0.537		
		Effective communication	0.197	0.254	0.014		
Multiple correlation – ‘stepwise’ method							
	FQoL	Constant	2.882		<0.001	0.407	<0.001
		Parents’ marital status	0.240	0.325	0.002		
		Family income	7.117e^-5^	0.316	0.002		
		Effective communication	0.210	0.270	0.007		
		Religion	0.189	0.228	0.025		

FQoL: Family Quality of Life.

## DISCUSSION

Our sample was characterized by better results in the “disability-related support” domain, which was expected because it was a convenience sample in which all families received support from APAE São Carlos. Schlebusch et al. also found that the “disability-related support” domain had the highest score. The explanation for their result also seems to apply to our study: their research was conducted among vulnerable families in South Africa who received disability-related support services, in a country where the scope of this kind of service is limited — a condition analogous to our sample. Thus, the high score of this domain would be explained by the fact that these families feel privileged and grateful^11^. This result is also compatible with studies conducted in Canada among both native and migrant families that demonstrated the importance for FQoL of access to external support^24,25^.

We consider that the higher scores of the “family interaction” and “parenting” domains can be explained by familism. Familism is a multidimensional construct that includes three dimensions operating within a family system: the structural dimension, which marks the spatial and social limits within which behaviors occur and attitudes acquire meaning (these limits are outlined by the presence or absence of family members); the attitudinal dimension, which refers to the expressed identification of family members with the interests and welfare of the family; and the behavioral dimension, which involves different degrees of attachment and affinity during contact between family members^26^. Familism is an especially important concept in families of Latin culture^27,28^, such as in Brazil, highly oriented by family values. In the context of social policies in Brazil, this configuration favors the family viewed as the main agent that offers goods and services for the welfare of individuals with disabilities, such that the family takes on most of the functions that should be the responsibility of the state^29,30^.

Based on our descriptive results, we consider that the marital status of the two parents can be a potential proxy for measuring the variability of the concept of familism. Our view is that among families in which the parents live together, this tends to translate into higher levels of familism than among families in which the parents live apart. In our study, the marital status of the two parents showed a significant relationship with the FQoL. Families in which the parents lived together, in comparison with families in which the parents lived apart, had higher average scores in the “family interaction” domain (3.99±0.34 *versus* 3.72±0.54; p=0.015) and in the “parenting” domain (3.85±0.25 *versus* 3.62±0.49; p=0.009). In the literature, it is suggested that this is a two-way phenomenon: on the one hand, not living together negatively impacts family relations; on the other hand, having a child with a disability implies higher divorce rates^31^.

The low score in the “emotional wellbeing” domain was consistent with findings in the literature^9,11,22,23,25,32–36^ and points to the criticality of emotional factors in FQoL in different cultures and social contexts. We consider that expansion of services offered by specialized professionals, such as psychologists and occupational therapists as well as organization of support groups for parents and guardians, would form viable solutions for this issue. Thus, initiatives that allow families more time to focus on issues that concern individuality and enable them to deal better with the daily stress of caregiving for a child with a disability are helpful.

The second domain that contributed to decreasing the FQoL in our sample, i.e. “physical/material wellbeing”, indicated that, in Brazil, policies for better income distribution, aimed at easing financial constraints among families with children with ASD and ID seem to be crucial. The other two studies conducted in Brazil using the BCFQoLS also showed that there were lower scores in the “physical/material wellbeing” domain than for the total FQoL^22,35^. In countries with advanced economies, however, the “physical/material wellbeing” domain has usually scored better^9,23,24,32^.

The family's financial health proved to be an important indicator of FQoL in our study. It was expressed in terms of three types of data: (1) household income range; (2) access to supplemental health insurance; and (3) a need to gain social benefits, while noting that the criterion for receive these benefits is, precisely, to have low income. Our correlation results between family income and FQoL were similar to those found in other studies^7,9,11^, in which family income was also a predictor of FQoL.

Two other factors were significantly related to the FQoL. Firstly, families that professed some religion had, on average, higher FQoL. We evaluated the influence of religion only by asking whether or not the family professed any religion, without considering other non-religious elements that comprise spirituality. Even considering the limits of our study, our results are consistent with those of another study that showed that religious practices contribute to increased resilience among people with disabilities^37^. Families that professed some religion reported having a sense of strength that was gained through spirituality and also built social ties with members of their religious community who, in turn, promoted acceptance of the child and their disability^38^. Given that spirituality plays an important role in an individual's quality of life, it is not surprising that religious practices could also influence the FQoL. Further exploratory analysis on this topic may result in important contributions to this field.

Secondly, our results also showed that the presence of effective communication among children with ID and ASD was significantly associated with higher FQoL scores. In a study conducted in Ireland, Fitzgerald et al. showed that the level of independence of children with ASD, including their communicative abilities, correlated with the family burden and influenced their mothers’ wellbeing^39^. Foley et al. compared families with children with Down syndrome whose communication skills were better and poorer and found results consistent with ours: the families in which the children had better communication skills had higher FQoL scores^40^. Our results also suggest that FQoL can be improved through actions that encourage proper communication by children with ID and ASD. However, testing this hypothesis would require an analytical study with a control group. We believe that our results reinforce the relevance of developing such an agenda.

This was the first Brazilian study to apply the BCFQoLS to a sample of families that have children with ID and ASD. We consider that the methodology used for data collection was a strength in our study: we conducted face-to-face interviews, which allowed us to clarify the participants’ doubts, thus increasing the reliability of the results. Moreover, use of validated instruments for classifying the degree of ID and ASD, and for assessing individuals’ functionality with regard to basic activities of daily living, made our results more objective and specific.

One limitation of our study concerned the data collection: data were only gathered from one family member, usually the mother, as done in most other research conducted in this area. Our results also presented bias because they reflected the specific reality of the sample and the scenario within which the study was developed. Furthermore, the set of correlational analyses, along with the associations between the variables presented in this study, should be considered with caution, given the nature of the research design. We believe that multicenter and analytical studies should be conducted to obtain a broad overview of the possible influences of socioenvironmental factors on the FQoL, which would enable formulation of public policies at the national level.

In conclusion, our results showed that the FQoL of the families investigated was sustained through factors such as family interaction and parents’ care for their children, and was negatively impacted by emotional wellbeing and physical and material conditions. We suggest that psychosocial support measures should be adopted in order to improve the emotional wellbeing of each family member, along with investments in social policies, material resources and human resources, so as to upgrade these families’ physical and material conditions and thus reduce their burden of caring for children with ID and ASD. Additionally, FQoL may also be improved through actions that encourage effective communication by children with ID and ASD.
